# Supplementary material for: Efficacy and safety of decompressive craniectomy with non-suture duraplasty in patients with traumatic brain injury
Source: PLoS One. 2020 Oct 8;15(10):e0232561. doi: 10.1371/journal.pone.0232561 (PMC7544056; doi:10.1371/journal.pone.0232561)
Supplement: Attachment — Submitted filename: Response to Reviewers.docx [file pone.0232561.s002.docx]

**Response to Reviewers**

Thank you for your sincere review of our paper. I fully agree with what you pointed out and have made several changes to this. The modified part is indicated in red. In addition, English correction was made again, and a certificate for this was attached.

#1: on page 2, line 30: “There were 37 patients in the non-suture group and 69 in the suture

craniectomy group” – I believe the sentence should be: “There were 37 patients in the non-suture duraplasty and 69 in the suture duraplasty group.”

It was modified as follows.

There were 37 patients in the non-suture duraplasty and 69 in the suture duraplasty group.

#2: on page 4, line 52: “Therefore, the purpose of this study was to determine if non-watertight dura reconstruction contributes significantly to the success of decompressive craniectomy in TBI” – I believe both techniques (with and without watertight dural closure) may lead to a successful decompressive craniectomy. The focus should be on the safety and feasibility of the non-watertight dural closure procedure.

As you pointed out, modifying it to safety and feasibility seems to be more appropriate for the purpose of the study. The technique used in our study was a non-suture duraplasty without suturing, and the contents were modified as follows.

Abstract - Background

“This study sought to determine the safety and feasibility of the non-suture dural closure technique in decompressive craniectomy.”

Introduction

“Therefore, the purpose of this study was to determine the safety and feasibility of non-suture duraplasty in the context of decompressive craniectomy for TBI patients.”

Conclusions

“Therefore, it can be assumed that non-suture duraplasty in decompressive craniectomy is a safe and feasible technique.”

#3: on page 5: the authors should clarify the definition of wound dehiscence. Also it is

important to explain how the intraoperative blood loss was measured. Furthermore, the most

feared complication of a non-watertight duraplasty decompressive craniectomy , CSF leak, is

not analyzed. If possible, the authors should include these data on their work.

The definition of wound dehiscence and the method of measuring intraoperative blood loss were added to the method. As you mentioned, we also believe that CSF leak is the most important complication of non-suture duraplasty. In our study, CSF leak was classified as follows. When CSF was discharged out of scalp through the surgical wound, it was classified as wound dehiscence or SSI according to the culture test result, and if CSF was discharged into the subcutaneous/epidural space but was not discharged outside the surgical wound, it was diagnosed as a subgaleal fluid collection. Because the definition of CSF leak may differ from reader to reader, CSF leak has been classified in detail. We have added this to the method. Also, the content of infection and mass effect that CSF leaks can cause has been added to the discussion.

Methods

“To quantify blood loss, an anesthesiologist measured the amount of fluid, including blood and irrigation fluid, collected through suction during surgery and then subtracted the amount of fluid used for irrigation from the measured volume. Final blood loss levels were also calculated based on the amount of blood on the gauze according to a method proposed by Ali Algadiem et al. [4].
Wound dehiscence was defined when a surgical incision reopened, without any discharge such as in the form of pus, and when bacteria were not identified from the opened site to distinguish superficial incisional SSIs. When CSF was drained through the surgical wound, it was determined whether it was a wound dehiscence or SSI, depending on the results of the culture test. When CSF was drained to the subcutaneous/epidural space, but not through the surgical wound, it was diagnosed as a subgaleal fluid collection.”

Discussion
“Subgaleal fluid collection was somewhat higher in the non-suture duraplasty group. Subgaleal fluid collection is thought to be mainly caused by CSF leaking out of the dura. It can cause trouble when fluid collection increases and incurs a massive effect, or when it works as a source of the SSI. Vieira et al. [3] reported that once the arachnoid is intact, there is no increased risk of CSF leakage, and watertight closures may lead to small defects on suture lines, causing a “one-way valve” effect that may potentially facilitate the development of CSF leakage. In suture duraplasty, CSF can be collected out of the dura unidirectionally using a one-way valve effect and can have a massive effect. However, in non-suture duraplasty, CSF is less likely to cause a massive effect by moving in and out of the dura. In patients who underwent decompressive craniectomy, it has been observed that the scalp of the craniectomy site swells up after coughing and gradually sinks as CSF moves in and out of the dura. In our study, only one brain CT scan per month after surgery was analyzed, meaning that only the condition at that time was effectively evaluated. It is necessary to evaluate the patterns of change of the subgaleal fluid collection through serial imaging analysis. With regard to the source of the SSI, subgaleal fluid collection is not likely to increase the SSI incidence because the SSI rate in the non-suture duraplasty group was lower than that of the suture duraplasty group.”

#4: on page 7: the treatment of epidural hematomas usually involves craniotomy, hematoma

evacuation, dural tenting sutures and fixation of the bone flap. In the majority of the cases, it

does not involve duraplasty and/or decompressive craniectomy. Yet, almost 15% of the

patients included in the study had an epidural hematoma. The authors need to clarify why

decompressive craniectomy was performed in this subset of patients.

Most traumatic brain injuries do not have only one finding, such as subdural or epidural hematoma, but are accompanied by various hemorrhagic findings. In our study, a representative finding was described as the diagnosis name. As you mentioned, for most epidural hematoma cases of our study, craniotomy without duraplasty was performed. However, if brain swelling/edema by contusions exists or is expected, craniectomy with duraplasty was performed. To clarify the reason for the duraplasty, we modified epidural hematoma in table 1 to epidural hematoma with contusion.

Table 1.

| Diagnosis |  |  |  | 0.269 |
| --- | --- | --- | --- | --- |
| Subdural hematoma | 55 (79.7) | 26 (70.3) | 81 (76.4) |  |
| Epidural hematoma with contusion | 7 (10.1) | 8 (21.6) | 15 (14.2) |  |
| Intracerebral hematoma | 7 (10.1) | 3 (8.1) | 10 (9.4) |  |

\# 5: on page 8: it is important to clarify how complications (subgaleal fluid collections,

hygromas, wound dehiscence and surgical site infections) were treated (if any treatment was

needed at all).

Thanks for the good point. We have added treatment methods for each complication to the discussion.

Discussion

“All cases of wound dehiscence were treated with a simple repair. The SSI was initially treated with antibiotics, which were selected according to the wound culture results. When the wound opens and pus is discharged, or when inflammation is not controlled despite the use of antibiotic therapy, surgical wound revision is carried out. In our study, four cases of suture duraplasty and two cases of non-suture duraplasty were treated with antibiotics. In addition, two cases of suture duraplasty were treated with antibiotics and surgical revision, including irrigation and granulation tissue removal, because pus was discharged from the opened wound.”

“In our study, all cases of subdural hygroma were observed without specific treatments because there were no findings linked to a massive effect or neurological symptoms caused by subdural hygroma, and most subdural hygromas resolved spontaneously. In addition, most patients with subgaleal fluid collection were closely observed because this did not incur a massive effect and was sometimes spontaneously absorbed. However, in two patients from the suture duraplasty group, subgaleal fluid collection increased gradually and compressed the brain, such that CSF leakage points were surgically repaired.”

\# 6: on page 10, line 159: “Surgical treatment is not always required, but may be necessary if it converts to a chronic subdural hematoma or is caused by a mass effect”- I believe hygromas should be treated if it causes mass effect.

We also believe that surgical treatment is necessary if subdural hygroma causes mass effects and neurological symptoms. We made the following changes.

Discussion

“Surgical treatment is not always required but is necessary if subdural hygroma causes a mass effect and becomes symptomatic.”

Reference.

*Paredes I, Cicuendez M, Delgado MA, Martinez-Pérez R, Munarriz PM, Lagares A. Normal pressure subdural hygroma with mass effect as a complication of decompressive craniectomy. Surg Neurol Int. 2011;2.*

\# 7: on page 10: in nearly 35% of the patients cranioplasty was performed. I believe it would

be more appropriate to include this data in the methods and results sections rather than just

in the discussion.

The content of cranioplasty has been added to the method and result sections.

Methods

“In addition, the results of cranioplasty, including intraoperative findings, GOS, and complications were reviewed, although the limited nature of the data meant that it could not be fully evaluated.”

Results

**Cranioplasty**

We observed 37 patients (suture duraplasty group: 27, non-suture duraplasty group: 10) who underwent cranioplasty following a decompressive craniectomy. GOS scores did not differ between the two groups (suture vs. non-suture), and patients with severe head injuries had a poorer prognosis. Therefore, we do not believe that watertight suturing necessarily leads to a good prognosis. In the non-suture duraplasty craniectomy group, the cortex was not exposed because of the formation of fibrotic tissue between the scalp flap and dura material (Fig 3). The incidence of complications, including infection after cranioplasty, were similar in the suture duraplasty and non-suture duraplasty groups (3% and 4%, respectively). Therefore, we believe that non-suture duraplasty may not cause any problems in cranioplasty, although further evaluation is warranted.
