# Supplementary material for: Efficacy and safety of decompressive craniectomy with non-suture duraplasty in patients with traumatic brain injury
Source: PLoS One. 2020 Oct 8;15(10):e0232561. doi: 10.1371/journal.pone.0232561 (PMC7544056; doi:10.1371/journal.pone.0232561)
Supplement: Attachment — Submitted filename: Response to Reviewers.docx [file pone.0232561.s003.docx]

**Response to Reviewers**

**Reviewer 1**

**# The incidence of CSF leak is unclear. Were all cases of wound dehiscence associated with CSF fistula? Were there cases of wound dehiscence without CSF fistula?**

CSF fistula can be defined as CSF leak outside the skin. In all cases of wound dehiscence, wound culture was carried out. If the bacteria were identified, it was diagnosed as SSI, and if the bacteria were not specified, it was diagnosed as wound dehiscence. In cases of wound dehiscence, it was difficult to accurately determine whether wound dehiscence was associated with CSF fistula because most of the medical records were not clearly specified or were described as simple discharge even if specified. We were not sure that the discharge was CSF, and we judged that determining whether the CSF fistula is present could lead to inaccurate results. So, we consisted of two items, wound dehiscence and SSI, for values of complication.

**# In table 1, regarding the diagnosis, I believe that the items "epidural hematoma with contusion" and "intracerebral hematoma" should be merged into a single item: "intracerebral contusion/hematoma". Epidural hematomas do not require decompressive craniectomy.**

We agree with your opinion, so we modified it to "intracerebral contusion/hematoma" as you pointed out.

**# Regarding cranioplasty, I think the data would be clearer if it was plotted in a table, similarly to Table 2. Furthermore, it is important to emphasize that the results session should only address study results. Phrases like "Therefore, we do not believe that watertight suturing necessarily leads to a good prognosis." should be reserved for the discussion or conclusions sessions.**

We made Table 3 of the cranioplasty results. However, because there were a few cases, it was difficult to obtain statistically significant results, so the p-value could not be calculated. Also, the incidence rate (3% vs. 4%) previously described was corrected by incorrectly indicating the incidence rate ratio (3:4) between the two groups.

**Cranioplasty**

We observed 37 patients (suture duraplasty group: 27, non-suture duraplasty group: 10) who underwent cranioplasty following a decompressive craniectomy. GOS scores did not differ between the two groups. And the incidence of complications, including infection after cranioplasty, were similar in the suture duraplasty and non-suture duraplasty groups (Table 3). In the non-suture duraplasty craniectomy group, the cortex was not exposed because of the formation of fibrotic tissue between the scalp flap and dura material (Fig 3).

**Table 3. Complications and GOS after cranioplasty**

|  | **Suture duraplasty (n=27)** | **Non-suture duraplasty (n=10)** |
| --- | --- | --- |
| Complications |  |  |
| Surgical site infection | 1 (3.7) | 0 (0) |
| Postoperative epidural hematoma | 1 (3.7) | 1 (10) |
| GOS score |  |  |
| 1 | 0 (0) | 0 (0) |
| 2 | 0 (0) | 0 (0) |
| 3 | 16 (59.3) | 6 (60) |
| 4 | 7 (25.9) | 2 (20) |
| 5 | 4 (14.8) | 2 (20) |

GOS: Glasgow Outcome Scale. Values are presented as numbers (%).

The phrase “Therefore ~” was deleted from the result session, and an additional paragraph was written in the discussion session.

**DISCUSSION**

Although there were not many cases, the results of cranioplasty showed that there was no significant difference between the two groups in GOS and complications. Therefore, we believe that non-suture duraplasty may not cause any problems in cranioplasty, although further evaluation is warranted.

**# Additionally, the text needs clarification on several points. I believe professional language editing service is needed. There are lots of them available on the internet. Actually, I believe that this is the main issue with the manuscript at the moment.**

We got a language editing service again. A certificate was also attached.

**Reviewer 2**

**# Why is age >70 years an exclusion criterion. What is the basis for excluding patients undergoing simultaneous surgery?**

Many studies have been reported that old age is one of the independent predictors of a worse outcome for traumatic brain injury. In comparing the prognosis according to the procedure method during craniectomy, we thought that the variable “age” could influence clinical outcomes such as GOS. Also, there are many cases of brain atrophy in elderly patients, and brain atrophy can affect the evaluation of CT findings such as subdural hygroma. For this reason, we excluded patients over 70 years of age from the study.

Simultaneous surgery is a surgery performed in multiple trauma patients (for example, when laparotomy and craniectomy are performed at the same time due to abdominal organ bleeding and TBI). Simultaneous surgery is very rare, with 2-3 cases a year, and most of the patients die due to DIC or hypotension that resulted from massive bleeding. There were 2 cases of simultaneous surgery during our study period. However, the patients died within one week, the postop brain CT required for the study results was not performed, and SSI was not evaluated, so simultaneous surgery was excluded from the study.
